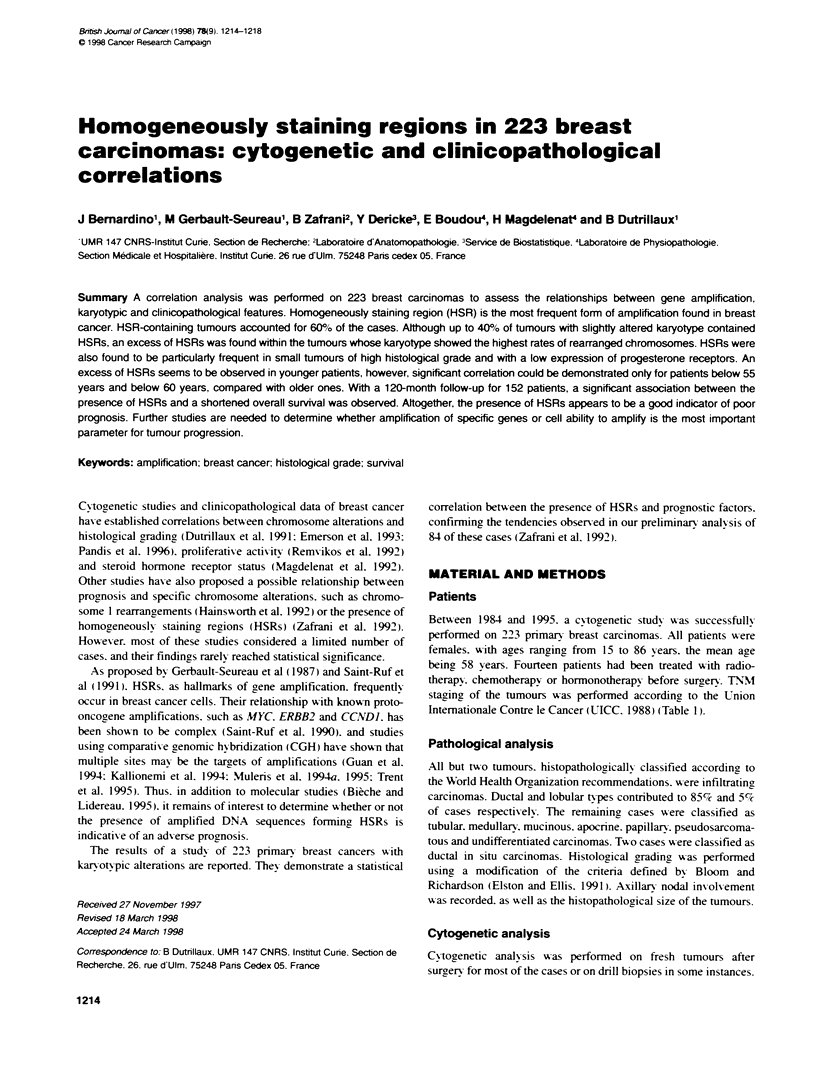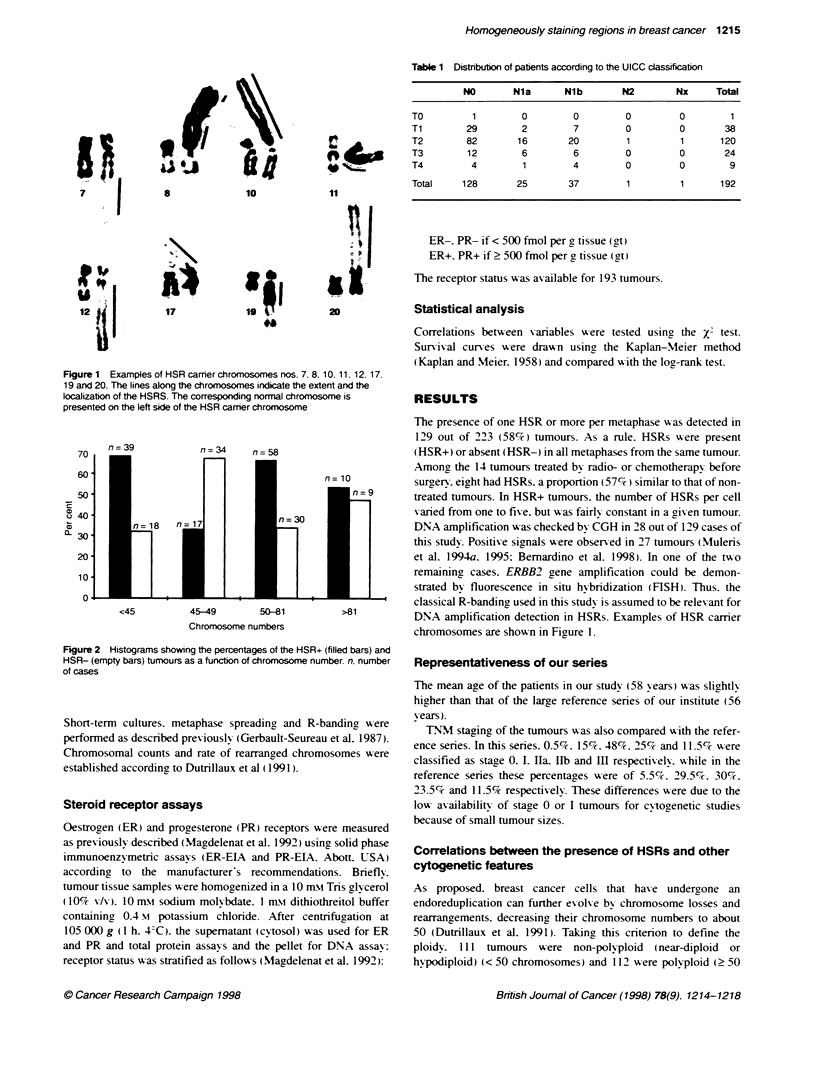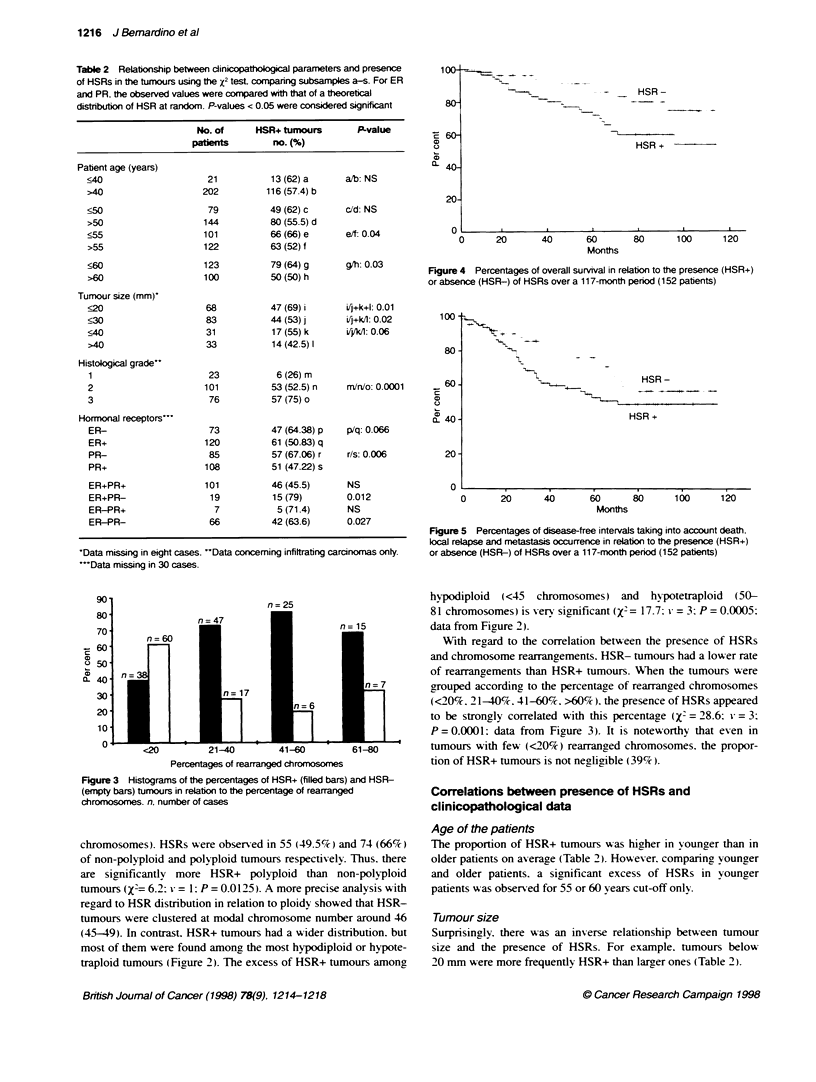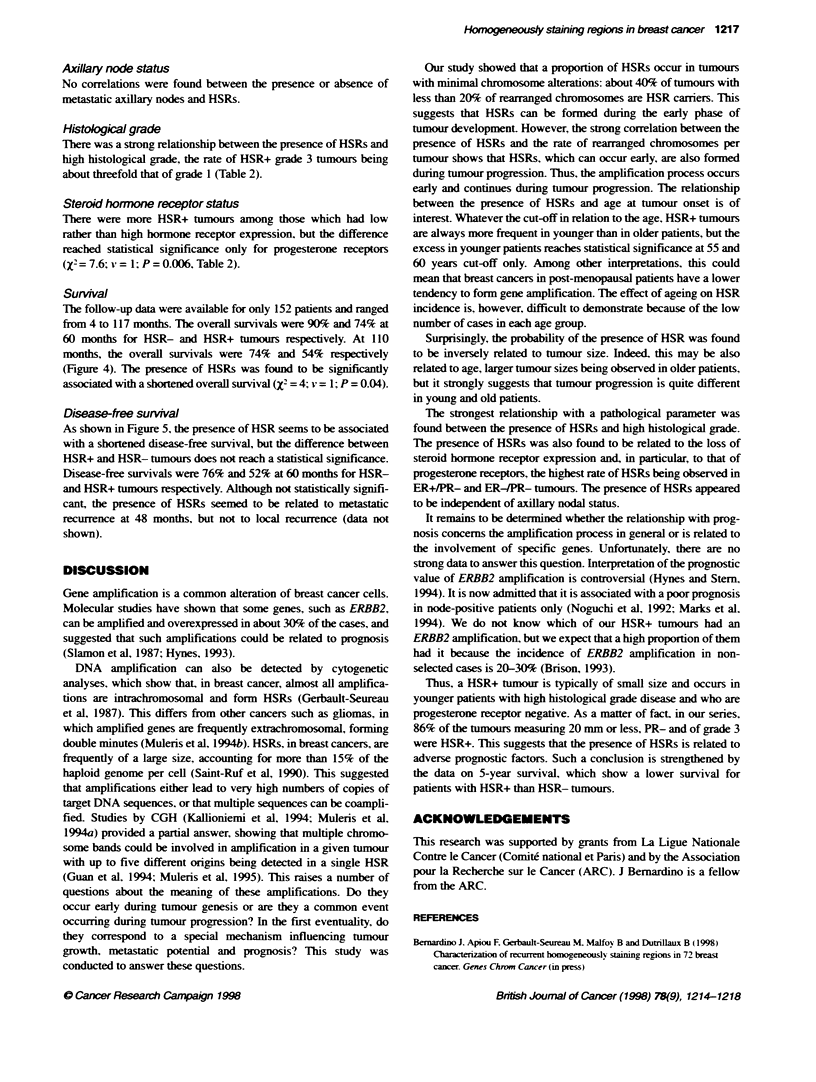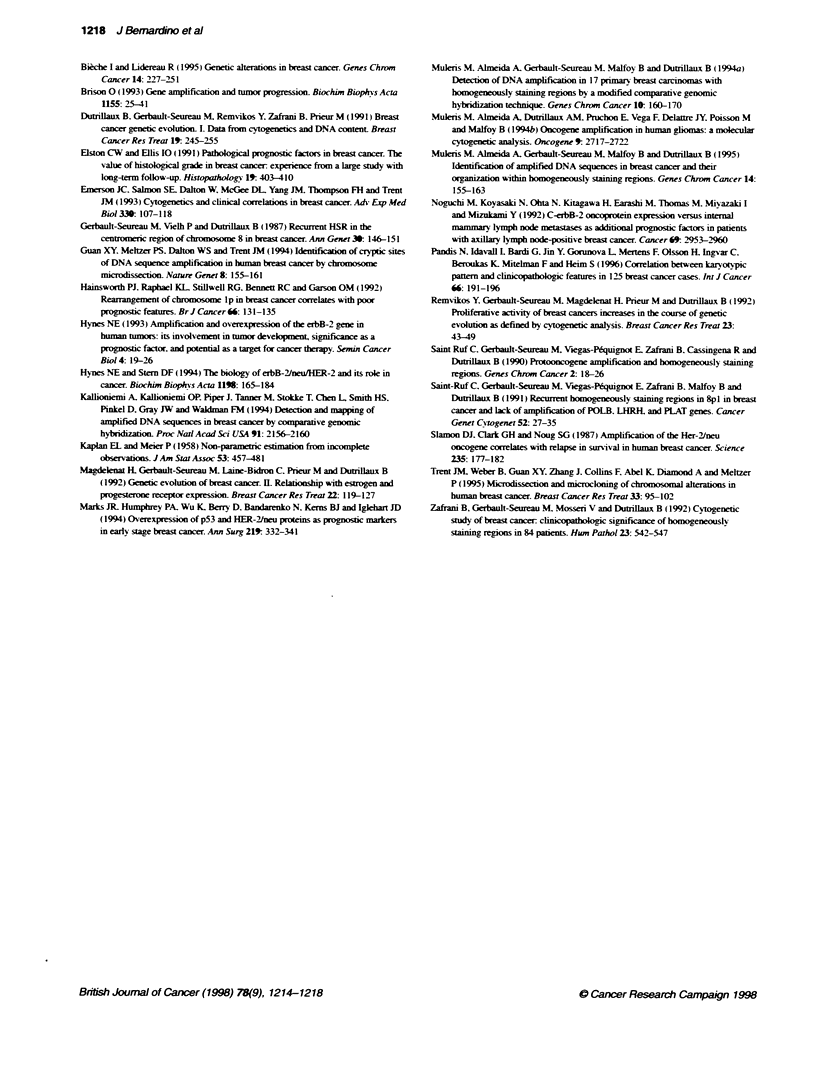# Homogeneously staining regions in 223 breast carcinomas: cytogenetic and clinicopathological correlations.

**DOI:** 10.1038/bjc.1998.657

**Published:** 1998-11

**Authors:** J. Bernardino, M. Gerbault-Seureau, B. Zafrani, Y. Dericke, E. Boudou, H. Magdelenat, B. Dutrillaux

**Affiliations:** UMR 147 CNRS-Institut Curie, Section de Recherche, Paris, France.

## Abstract

**Images:**


British Journal of Cancer (1998) 78(9). 1214–1218
© 1998 Cancer Research Campaign

### Homogeneously staining regions in 223 breast carcinomas: cytogenetic and clinicopathological correlations

**J Bernardino[1], M Gerbault-Seureau[1], B Zafrani[2], Y Dericke[3], E Boudou[4], H Magdelenat[4] and B Dutrillaux[1]**

[1]UMR 147 CNRS-Institut Curie. Section de Recherche: [2]Laboratoire d'Anatomopathologie. [3]Service de Biostatistique. [4]Laboratoire de Physiopathologie. Section Médicale et Hospitalière. Institut Curie. 26 rue d'Ulm. 75248 Paris cedex 05. France

**Summary** A correlation analysis was performed on 223 breast carcinomas to assess the relationships between gene amplification, karyotypic and clinicopathological features. Homogeneously staining region (HSR) is the most frequent form of amplification found in breast cancer. HSR-containing tumours accounted for 60% of the cases. Although up to 40% of tumours with slightly altered karyotype contained HSRs, an excess of HSRs was found within the tumours whose karyotype showed the highest rates of rearranged chromosomes. HSRs were also found to be particularly frequent in small tumours of high histological grade and with a low expression of progesterone receptors. An excess of HSRs seems to be observed in younger patients, however, significant correlation could be demonstrated only for patients below 55 years and below 60 years, compared with older ones. With a 120-month follow-up for 152 patients, a significant association between the presence of HSRs and a shortened overall survival was observed. Altogether, the presence of HSRs appears to be a good indicator of poor prognosis. Further studies are needed to determine whether amplification of specific genes or cell ability to amplify is the most important parameter for tumour progression.

**Keywords:** amplification; breast cancer; histological grade; survival

Cytogenetic studies and clinicopathological data of breast cancer have established correlations between chromosome alterations and histological grading (Dutrillaux et al. 1991: Emerson et al. 1993: Pandis et al. 1996). proliferative activity (Remvikos et al. 1992) and steroid hormone receptor status (Magdelenat et al. 1992). Other studies have also proposed a possible relationship between prognosis and specific chromosome alterations. such as chromosome 1 rearrangements (Hainsworth et al. 1992) or the presence of homogeneously staining regions (HSRs) (Zafrani et al. 1992). However. most of these studies considered a limited number of cases. and their findings rarely reached statistical significance.

As proposed by Gerbault-Seureau et al (1987) and Saint-Ruf et al (1991). HSRs. as hallmarks of gene amplification. frequently occur in breast cancer cells. Their relationship with known proto-oncogene amplifications. such as *MYC*. *ERBB2* and *CCND1*. has been shown to be complex (Saint-Ruf et al. 1990). and studies using comparative genomic hybridization (CGH) have shown that multiple sites may be the targets of amplifications (Guan et al. 1994: Kallionemi et al. 1994: Muleris et al. 1994*a*. 1995: Trent et al. 1995). Thus. in addition to molecular studies (Bièche and Lidereau. 1995). it remains of interest to determine whether or not the presence of amplified DNA sequences forming HSRs is indicative of an adverse prognosis.

The results of a study of 223 primary breast cancers with karyotypic alterations are reported. They demonstrate a statistical correlation between the presence of HSRs and prognostic factors. confirming the tendencies observed in our preliminary analysis of 84 of these cases (Zafrani et al. 1992).

*Received 27 November 1997*
*Revised 18 March 1998*
*Accepted 24 March 1998*

*Correspondence to:* B Dutrillaux. UMR 147 CNRS. Institut Curie. Section de Recherche. 26. rue d'Ulm. 75248 Paris Cedex 05. France

### MATERIAL AND METHODS

### Patients

Between 1984 and 1995. a cytogenetic study was successfully performed on 223 primary breast carcinomas. All patients were females. with ages ranging from 15 to 86 years. the mean age being 58 years. Fourteen patients had been treated with radiotherapy. chemotherapy or hormonotherapy before surgery. TNM staging of the tumours was performed according to the Union Internationale Contre le Cancer (UICC. 1988) (Table 1).

### Pathological analysis

All but two tumours. histopathologically classified according to the World Health Organization recommendations. were infiltrating carcinomas. Ductal and lobular types contributed to 85% and 5% of cases respectively. The remaining cases were classified as tubular. medullary. mucinous. apocrine. papillary. pseudosarcomatous and undifferentiated carcinomas. Two cases were classified as ductal in situ carcinomas. Histological grading was performed using a modification of the criteria defined by Bloom and Richardson (Elston and Ellis. 1991). Axillary nodal involvement was recorded. as well as the histopathological size of the tumours.

### Cytogenetic analysis

Cytogenetic analysis was performed on fresh tumours after surgery for most of the cases or on drill biopsies in some instances.

**Figure 1** Examples of HSR carrier chromosomes nos. 7. 8. 10. 11. 12. 17. 19 and 20. The lines along the chromosomes indicate the extent and the localization of the HSRS. The corresponding normal chromosome is presented on the left side of the HSR carrier chromosome

**Figure 2** Histograms showing the percentages of the HSR+ (filled bars) and HSR– (empty bars) tumours as a function of chromosome number. *n*. number of cases

Short-term cultures. metaphase spreading and R-banding were performed as described previously (Gerbault-Seureau et al. 1987). Chromosomal counts and rate of rearranged chromosomes were established according to Dutrillaux et al (1991).

### Steroid receptor assays

Oestrogen (ER) and progesterone (PR) receptors were measured as previously described (Magdelenat et al. 1992) using solid phase immunoenzymetric assays (ER-EIA and PR-EIA. Abott. USA) according to the manufacturer's recommendations. Briefly. tumour tissue samples were homogenized in a 10 mM Tris glycerol (10% v/v). 10 mM sodium molybdate. 1 mM dithiothreitol buffer containing 0.4 M potassium chloride. After centrifugation at 105 000 *g* (1 h. 4°C). the supernatant (cytosol) was used for ER and PR and total protein assays and the pellet for DNA assay: receptor status was stratified as follows (Magdelenat et al. 1992):

**Table 1** Distribution of patients according to the UICC classification

| | N0 | N1a | N1b | N2 | Nx | Total |
|---|---|---|---|---|---|---|
| T0 | 1 | 0 | 0 | 0 | 0 | 1 |
| T1 | 29 | 2 | 7 | 0 | 0 | 38 |
| T2 | 82 | 16 | 20 | 1 | 1 | 120 |
| T3 | 12 | 6 | 6 | 0 | 0 | 24 |
| T4 | 4 | 1 | 4 | 0 | 0 | 9 |
| Total | 128 | 25 | 37 | 1 | 1 | 192 |

ER–. PR– if < 500 fmol per g tissue (gt)
ER+. PR+ if ≥ 500 fmol per g tissue (gt)

The receptor status was available for 193 tumours.

### Statistical analysis

Correlations between variables were tested using the $\chi^2$ test. Survival curves were drawn using the Kaplan–Meier method (Kaplan and Meier. 1958) and compared with the log-rank test.

### RESULTS

The presence of one HSR or more per metaphase was detected in 129 out of 223 (58%) tumours. As a rule. HSRs were present (HSR+) or absent (HSR–) in all metaphases from the same tumour. Among the 14 tumours treated by radio- or chemotherapy before surgery. eight had HSRs. a proportion (57%) similar to that of non-treated tumours. In HSR+ tumours. the number of HSRs per cell varied from one to five. but was fairly constant in a given tumour. DNA amplification was checked by CGH in 28 out of 129 cases of this study. Positive signals were observed in 27 tumours (Muleris et al. 1994*a*. 1995: Bernardino et al. 1998). In one of the two remaining cases. *ERBB2* gene amplification could be demonstrated by fluorescence in situ hybridization (FISH). Thus. the classical R-banding used in this study is assumed to be relevant for DNA amplification detection in HSRs. Examples of HSR carrier chromosomes are shown in Figure 1.

### Representativeness of our series

The mean age of the patients in our study (58 years) was slightly higher than that of the large reference series of our institute (56 years).

TNM staging of the tumours was also compared with the reference series. In this series. 0.5%. 15%. 48%. 25% and 11.5% were classified as stage 0. I. IIa. IIb and III respectively. while in the reference series these percentages were of 5.5%. 29.5%. 30%. 23.5% and 11.5% respectively. These differences were due to the low availability of stage 0 or I tumours for cytogenetic studies because of small tumour sizes.

### Correlations between the presence of HSRs and other cytogenetic features

As proposed. breast cancer cells that have undergone an endoreduplication can further evolve by chromosome losses and rearrangements. decreasing their chromosome numbers to about 50 (Dutrillaux et al. 1991). Taking this criterion to define the ploidy. 111 tumours were non-polyploid (near-diploid or hypodiploid) (< 50 chromosomes) and 112 were polyploid (≥ 50

© Cancer Research Campaign 1998

**Table 2** Relationship between clinicopathological parameters and presence of HSRs in the tumours using the $\chi^2$ test, comparing subsamples a–s. For ER and PR, the observed values were compared with that of a theoretical distribution of HSR at random. P-values < 0.05 were considered significant

| | No. of patients | HSR+ tumours no. (%) | P-value |
|---|---|---|---|
| Patient age (years) | | | |
| ≤40 | 21 | 13 (62) a | a/b: NS |
| >40 | 202 | 116 (57.4) b | |
| ≤50 | 79 | 49 (62) c | c/d: NS |
| >50 | 144 | 80 (55.5) d | |
| ≤55 | 101 | 66 (66) e | e/f: 0.04 |
| >55 | 122 | 63 (52) f | |
| ≤60 | 123 | 79 (64) g | g/h: 0.03 |
| >60 | 100 | 50 (50) h | |
| Tumour size (mm)* | | | |
| ≤20 | 68 | 47 (69) i | i/j+k+l: 0.01 |
| ≤30 | 83 | 44 (53) j | i/j+k/l: 0.02 |
| ≤40 | 31 | 17 (55) k | i/j/k/l: 0.06 |
| >40 | 33 | 14 (42.5) l | |
| Histological grade** | | | |
| 1 | 23 | 6 (26) m | |
| 2 | 101 | 53 (52.5) n | m/n/o: 0.0001 |
| 3 | 76 | 57 (75) o | |
| Hormonal receptors*** | | | |
| ER– | 73 | 47 (64.38) p | p/q: 0.066 |
| ER+ | 120 | 61 (50.83) q | |
| PR– | 85 | 57 (67.06) r | r/s: 0.006 |
| PR+ | 108 | 51 (47.22) s | |
| ER+PR+ | 101 | 46 (45.5) | NS |
| ER+PR– | 19 | 15 (79) | 0.012 |
| ER–PR+ | 7 | 5 (71.4) | NS |
| ER–PR– | 66 | 42 (63.6) | 0.027 |

*Data missing in eight cases. **Data concerning infiltrating carcinomas only. ***Data missing in 30 cases.

**Figure 3** Histograms of the percentages of HSR+ (filled bars) and HSR– (empty bars) tumours in relation to the percentage of rearranged chromosomes. n, number of cases chromosomes). HSRs were observed in 55 (49.5%) and 74 (66%) of non-polyploid and polyploid tumours respectively. Thus, there are significantly more HSR+ polyploid than non-polyploid tumours ($\chi^2$= 6.2; $v$ = 1; $P$ = 0.0125). A more precise analysis with regard to HSR distribution in relation to ploidy showed that HSR– tumours were clustered at modal chromosome number around 46 (45–49). In contrast, HSR+ tumours had a wider distribution, but most of them were found among the most hypodiploid or hypotetraploid tumours (Figure 2). The excess of HSR+ tumours among

**Figure 4** Percentages of overall survival in relation to the presence (HSR+) or absence (HSR–) of HSRs over a 117-month period (152 patients)

**Figure 5** Percentages of disease-free intervals taking into account death, local relapse and metastasis occurrence in relation to the presence (HSR+) or absence (HSR–) of HSRs over a 117-month period (152 patients)

hypodiploid (<45 chromosomes) and hypotetraploid (50–81 chromosomes) is very significant ($\chi^2$= 17.7; $v$ = 3; $P$ = 0.0005; data from Figure 2).

With regard to the correlation between the presence of HSRs and chromosome rearrangements, HSR– tumours had a lower rate of rearrangements than HSR+ tumours. When the tumours were grouped according to the percentage of rearranged chromosomes (<20%, 21–40%, 41–60%, >60%), the presence of HSRs appeared to be strongly correlated with this percentage ($\chi^2$ = 28.6; $v$ = 3; $P$ = 0.0001; data from Figure 3). It is noteworthy that even in tumours with few (<20%) rearranged chromosomes, the proportion of HSR+ tumours is not negligible (39%).

### Correlations between presence of HSRs and clinicopathological data

### *Age of the patients*

The proportion of HSR+ tumours was higher in younger than in older patients on average (Table 2). However, comparing younger and older patients, a significant excess of HSRs in younger patients was observed for 55 or 60 years cut-off only.

### *Tumour size*

Surprisingly, there was an inverse relationship between tumour size and the presence of HSRs. For example, tumours below 20 mm were more frequently HSR+ than larger ones (Table 2).

© Cancer Research Campaign 1998

### *Axillary node status*

No correlations were found between the presence or absence of metastatic axillary nodes and HSRs.

### *Histological grade*

There was a strong relationship between the presence of HSRs and high histological grade, the rate of HSR+ grade 3 tumours being about threefold that of grade 1 (Table 2).

### *Steroid hormone receptor status*

There were more HSR+ tumours among those which had low rather than high hormone receptor expression, but the difference reached statistical significance only for progesterone receptors ($\chi^2 = 7.6$; $v = 1$; $P = 0.006$, Table 2).

### *Survival*

The follow-up data were available for only 152 patients and ranged from 4 to 117 months. The overall survivals were 90% and 74% at 60 months for HSR– and HSR+ tumours respectively. At 110 months, the overall survivals were 74% and 54% respectively (Figure 4). The presence of HSRs was found to be significantly associated with a shortened overall survival ($\chi^2 = 4$; $v = 1$; $P = 0.04$).

### *Disease-free survival*

As shown in Figure 5, the presence of HSR seems to be associated with a shortened disease-free survival, but the difference between HSR+ and HSR– tumours does not reach a statistical significance. Disease-free survivals were 76% and 52% at 60 months for HSR– and HSR+ tumours respectively. Although not statistically significant, the presence of HSRs seemed to be related to metastatic recurrence at 48 months, but not to local recurrence (data not shown).

### DISCUSSION

Gene amplification is a common alteration of breast cancer cells. Molecular studies have shown that some genes, such as *ERBB2*, can be amplified and overexpressed in about 30% of the cases, and suggested that such amplifications could be related to prognosis (Slamon et al, 1987; Hynes, 1993).

DNA amplification can also be detected by cytogenetic analyses, which show that, in breast cancer, almost all amplifications are intrachromosomal and form HSRs (Gerbault-Seureau et al, 1987). This differs from other cancers such as gliomas, in which amplified genes are frequently extrachromosomal, forming double minutes (Muleris et al, 1994*b*). HSRs, in breast cancers, are frequently of a large size, accounting for more than 15% of the haploid genome per cell (Saint-Ruf et al, 1990). This suggested that amplifications either lead to very high numbers of copies of target DNA sequences, or that multiple sequences can be coamplified. Studies by CGH (Kallioniemi et al, 1994; Muleris et al, 1994*a*) provided a partial answer, showing that multiple chromosome bands could be involved in amplification in a given tumour with up to five different origins being detected in a single HSR (Guan et al, 1994; Muleris et al, 1995). This raises a number of questions about the meaning of these amplifications. Do they occur early during tumour genesis or are they a common event occurring during tumour progression? In the first eventuality, do they correspond to a special mechanism influencing tumour growth, metastatic potential and prognosis? This study was conducted to answer these questions.

Our study showed that a proportion of HSRs occur in tumours with minimal chromosome alterations: about 40% of tumours with less than 20% of rearranged chromosomes are HSR carriers. This suggests that HSRs can be formed during the early phase of tumour development. However, the strong correlation between the presence of HSRs and the rate of rearranged chromosomes per tumour shows that HSRs, which can occur early, are also formed during tumour progression. Thus, the amplification process occurs early and continues during tumour progression. The relationship between the presence of HSRs and age at tumour onset is of interest. Whatever the cut-off in relation to the age, HSR+ tumours are always more frequent in younger than in older patients, but the excess in younger patients reaches statistical significance at 55 and 60 years cut-off only. Among other interpretations, this could mean that breast cancers in post-menopausal patients have a lower tendency to form gene amplification. The effect of ageing on HSR incidence is, however, difficult to demonstrate because of the low number of cases in each age group.

Surprisingly, the probability of the presence of HSR was found to be inversely related to tumour size. Indeed, this may be also related to age, larger tumour sizes being observed in older patients, but it strongly suggests that tumour progression is quite different in young and old patients.

The strongest relationship with a pathological parameter was found between the presence of HSRs and high histological grade. The presence of HSRs was also found to be related to the loss of steroid hormone receptor expression and, in particular, to that of progesterone receptors, the highest rate of HSRs being observed in ER+/PR– and ER–/PR– tumours. The presence of HSRs appeared to be independent of axillary nodal status.

It remains to be determined whether the relationship with prognosis concerns the amplification process in general or is related to the involvement of specific genes. Unfortunately, there are no strong data to answer this question. Interpretation of the prognostic value of *ERBB2* amplification is controversial (Hynes and Stern, 1994). It is now admitted that it is associated with a poor prognosis in node-positive patients only (Noguchi et al, 1992; Marks et al, 1994). We do not know which of our HSR+ tumours had an *ERBB2* amplification, but we expect that a high proportion of them had it because the incidence of *ERBB2* amplification in non-selected cases is 20–30% (Brison, 1993).

Thus, a HSR+ tumour is typically of small size and occurs in younger patients with high histological grade disease and who are progesterone receptor negative. As a matter of fact, in our series, 86% of the tumours measuring 20 mm or less, PR– and of grade 3 were HSR+. This suggests that the presence of HSRs is related to adverse prognostic factors. Such a conclusion is strengthened by the data on 5-year survival, which show a lower survival for patients with HSR+ than HSR– tumours.

### ACKNOWLEDGEMENTS

This research was supported by grants from La Ligue Nationale Contre le Cancer (Comité national et Paris) and by the Association pour la Recherche sur le Cancer (ARC). J Bernardino is a fellow from the ARC.

### REFERENCES

Bernardino J, Apiou F, Gerbault-Seureau M, Malfoy B and Dutrillaux B (1998) Characterization of recurrent homogeneously staining regions in 72 breast cancer. *Genes Chrom Cancer* (in press)

© Cancer Research Campaign 1998

Bièche I and Lidereau R (1995) Genetic alterations in breast cancer. *Genes Chrom Cancer* **14**: 227–251

Brison O (1993) Gene amplification and tumor progression. *Biochim Biophys Acta* **1155**: 25–41

Dutrillaux B, Gerbault-Seureau M, Remvikos Y, Zafrani B, Prieur M (1991) Breast cancer genetic evolution. I. Data from cytogenetics and DNA content. *Breast Cancer Res Treat* **19**: 245–255

Elston CW and Ellis IO (1991) Pathological prognostic factors in breast cancer. The value of histological grade in breast cancer: experience from a large study with long-term follow-up. *Histopathology* **19**: 403–410

Emerson JC, Salmon SE, Dalton W, McGee DL, Yang JM, Thompson FH and Trent JM (1993) Cytogenetics and clinical correlations in breast cancer. *Adv Exp Med Biol* **330**: 107–118

Gerbault-Seureau M, Vielh P and Dutrillaux B (1987) Recurrent HSR in the centromeric region of chromosome 8 in breast cancer. *Ann Genet* **30**: 146–151

Guan XY, Meltzer PS, Dalton WS and Trent JM (1994) Identification of cryptic sites of DNA sequence amplification in human breast cancer by chromosome microdissection. *Nature Genet* **8**: 155–161

Hainsworth PJ, Raphael KL, Stillwell RG, Bennett RC and Garson OM (1992) Rearrangement of chromosome 1p in breast cancer correlates with poor prognostic features. *Br J Cancer* **66**: 131–135

Hynes NE (1993) Amplification and overexpression of the erbB-2 gene in human tumors: its involvement in tumor development, significance as a prognostic factor, and potential as a target for cancer therapy. *Semin Cancer Biol* **4**: 19–26

Hynes NE and Stern DF (1994) The biology of erbB-2/neu/HER-2 and its role in cancer. *Biochim Biophys Acta* **1198**: 165–184

Kallioniemi A, Kallioniemi OP, Piper J, Tanner M, Stokke T, Chen L, Smith HS, Pinkel D, Gray JW and Waldman FM (1994) Detection and mapping of amplified DNA sequences in breast cancer by comparative genomic hybridization. *Proc Natl Acad Sci USA* **91**: 2156–2160

Kaplan EL and Meier P (1958) Non-parametric estimation from incomplete observations. *J Am Stat Assoc* **53**: 457–481

Magdelenat H, Gerbault-Seureau M, Laine-Bidron C, Prieur M and Dutrillaux B (1992) Genetic evolution of breast cancer. II. Relationship with estrogen and progesterone receptor expression. *Breast Cancer Res Treat* **22**: 119–127

Marks JR, Humphrey PA, Wu K, Berry D, Bandarenko N, Kerns BJ and Iglehart JD (1994) Overexpression of p53 and HER-2/neu proteins as prognostic markers in early stage breast cancer. *Ann Surg* **219**: 332–341

Muleris M, Almeida A, Gerbault-Seureau M, Malfoy B and Dutrillaux B (1994*a*) Detection of DNA amplification in 17 primary breast carcinomas with homogeneously staining regions by a modified comparative genomic hybridization technique. *Genes Chrom Cancer* **10**: 160–170

Muleris M, Almeida A, Dutrillaux AM, Pruchon E, Vega F, Delattre JY, Poisson M and Malfoy B (1994*b*) Oncogene amplification in human gliomas: a molecular cytogenetic analysis. *Oncogene* **9**: 2717–2722

Muleris M, Almeida A, Gerbault-Seureau M, Malfoy B and Dutrillaux B (1995) Identification of amplified DNA sequences in breast cancer and their organization within homogeneously staining regions. *Genes Chrom Cancer* **14**: 155–163

Noguchi M, Koyasaki N, Ohta N, Kitagawa H, Earashi M, Thomas M, Miyazaki I and Mizukami Y (1992) C-erbB-2 oncoprotein expression versus internal mammary lymph node metastases as additional prognostic factors in patients with axillary lymph node-positive breast cancer. *Cancer* **69**: 2953–2960

Pandis N, Idavall I, Bardi G, Jin Y, Gorunova L, Mertens F, Olsson H, Ingvar C, Beroukas K, Mitelman F and Heim S (1996) Correlation between karyotypic pattern and clinicopathologic features in 125 breast cancer cases. *Int J Cancer* **66**: 191–196

Remvikos Y, Gerbault-Seureau M, Magdelenat H, Prieur M and Dutrillaux B (1992) Proliferative activity of breast cancers increases in the course of genetic evolution as defined by cytogenetic analysis. *Breast Cancer Res Treat* **23**: 43–49

Saint Ruf C, Gerbault-Seureau M, Viegas-Péquignot E, Zafrani B, Cassingena R and Dutrillaux B (1990) Protooncogene amplification and homogeneously staining regions. *Genes Chrom Cancer* **2**: 18–26

Saint-Ruf C, Gerbault-Seureau M, Viegas-Péquignot E, Zafrani B, Malfoy B and Dutrillaux B (1991) Recurrent homogeneously staining regions in 8p1 in breast cancer and lack of amplification of POLB, LHRH, and PLAT genes. *Cancer Genet Cytogenet* **52**: 27–35

Slamon DJ, Clark GH and Noug SG (1987) Amplification of the Her-2/neu oncogene correlates with relapse in survival in human breast cancer. *Science* **235**: 177–182

Trent JM, Weber B, Guan XY, Zhang J, Collins F, Abel K, Diamond A and Meltzer P (1995) Microdissection and microcloning of chromosomal alterations in human breast cancer. *Breast Cancer Res Treat* **33**: 95–102

Zafrani B, Gerbault-Seureau M, Mosseri V and Dutrillaux B (1992) Cytogenetic study of breast cancer: clinicopathologic significance of homogeneously staining regions in 84 patients. *Hum Pathol* **23**: 542–547

© Cancer Research Campaign 1998